# What is the role of the thioredoxin antioxidant complex in relation to HAM/TSP?

**DOI:** 10.1099/acmi.0.000090

**Published:** 2020-01-14

**Authors:** Masoud Youssefi, Kiarash Ghazvini, Jorge Casseb, Masoud keikha

**Affiliations:** ^1^​ Antimicrobial Resistance Research Center, Mashhad University of Medical Sciences, Mashhad, Iran; ^2^​ Department of Microbiology and Virology, Faculty of Medicine, Mashhad University of Medical Sciences, Mashhad, Iran; ^3^​ Department of Dermatology, Institute of Tropical Medicine of São Paulo/ Laboratory of Dermatology and Immunodeficiencies, University of São Paulo Medical School, São Paulo, SP 01246-100, Brazil

**Keywords:** HTLV-1, HAM/TSP, infection, thioredoxin reductase

## Abstract

We have little information about the definite role of the thioredoxin antioxidant complex system during viral infection, particularly during human T-cell lymphotropic virus type 1 (HTLV-1) infection and the HTLV-1-associated myelopathy/tropical spastic paraparesis (HAM/TSP) state. Therefore, we conducted comprehensive next-generation sequencing (NGS) analysis to determine Trx system expression changes in three categories of subjects: sero-negative HTLV-1 individuals, asymptomatic HTLV-1 people and HAM/TSP patients. We found that Trx capacity is reduced in the HAM/TSP state compared to healthy individuals, which indicates increasing inflammation and reduction of apoptosis, which might contribute to the progression of inflammation in the spinal cord, which in turn may develop into the HAM/TSP state.

## Introduction

Human T-cell lymphotropic virus type 1 (HTLV-1) is one of the most important retrovirus type C members, affecting 5–10 million people worldwide [1]. This virus is endemic in many areas, including Japan, sub-Saharan Africa, the Caribbean islands, South America, the Middle East (particularly northeastern Iran), Romania and Australo-Melanesia [1, 2].

HTLV-1 is the aetiological agent of serious disorders such as HTLV-1-associated myelopathy/tropical spastic paraparesis (HAM/TSP) and adult T-cell leukaemia/lymphoma (ATLL) [3]. Although over 90 % of HTLV-1-infected people remain asymptomatic carriers throughout their lives, approximately 1–2 % of infected individuals develop HAM/TSP and another 2–4% develop the fatal hematological condition of ATLL [2, 3]. The precise mechanisms of HAM/TSP have not yet been fully elucidated. However, increasing proviral load, viral virulence factors such as Tax, HBZ, Rex, p12 and p30/13, and host epigenetic changes, in particular dysregulation and modulation of the immune system, may be predisposing factors for these disorders [1–3]. In addition, there is now growing evidence for the increased likelihood of progression to HAM/TSP in the Caribbean and South American populations and those infected with the virus through contaminated blood products or unsafe sexual contacts [2, 3].

The most important hallmarks of HAM/TSP include chronic inflammation in the cerebro-spinal fluid (CSF); increased plasma levels of pro-inflammatory cytokines (i.e. IL-4, IL-6, IL-8, IFN-ϒ and TNF-α); CD4+ T cells, which continuously and spontaneously produce IFN- ϒ, TNF-α, IL-6 and IL-1β; and increased levels of CXCL10, CXCL9 and CXCR3 in the CSF, as well as numerous indicators of chronic inflammation in the CSF [2, 4]. In relation to the pathogenesis of HAM/TSP, it has been suggested that HTLV-1-infected CD4+ T cells spontaneously produce large quantities of IFN-ϒ and pro-inflammatory cytokines, causing tight junction disruption of the blood–brain barrier and leading to the exacerbation of inflammation in the CSF. Subsequently, the glial central nervous system (CNS) cell astrocytes are stimulated in response to the increased IFN-ϒ and secrete the chemokines CXCL9 and CXCL10, which cause the migration and recruitment of the CD4+ and CTLs to the CSF. Finally, clinical manifestations appear along with a prolonged inflammation process and demyelination and axonal loss of the neural cells [2, 4, 5]. One of the mechanisms through which inflammation causes cell damage and disease exacerbation is the production of free radicals and oxidative stress. In turn, the human body employs several mechanisms against HTLV-1 infection to reduce free radical damage (iNOS) or oxidative stress [6].

The Trx system is one of the most important antioxidant systems in the body that protects the cells from degradation due to oxidative stress and also inhibits apoptosis through AP-1, NF_κB and AMPK [7, 8]. According to a review of the literature, CD4+ T cells cells produce large amounts of Trx during retroviral infections, particularly in HIV and HTLV-1 infection. This would reduce the damage due to oxidative stress and is considered to be one of the potential therapeutic alternatives for HTLV-1, working by reducing the release of inflammatory cytokines [9–14]. However, the role of the Trx system in HTLV-1 pathogenesis has not yet been investigated and quite limited studies have been performed.

The aim of this study was to evaluate the expression changes of Trx system genes (including Trx, Trx reductase, NADPH and scavenger ROS) and some of the most important genes responsible for T cell proliferation and apoptosis.

## Methods

**Fig. 1.**
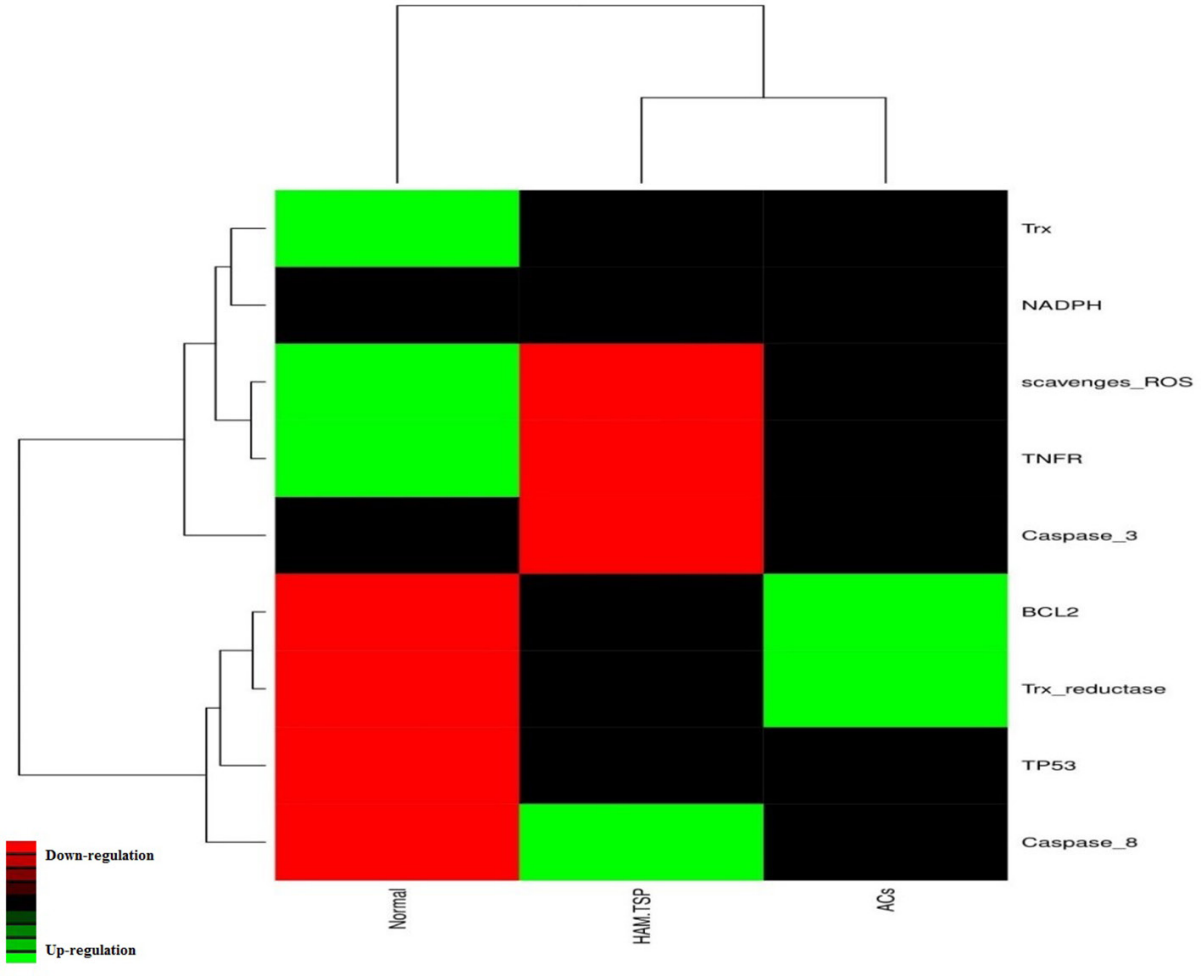
Fig. 1: Heatmap of the hub genes in different patient categories (healthy, ACs and HAM/TSP). The colour of the genes indicates the expression level. Red represents a lower expression level while overexpression is indicated using green.

First, all the HTLV-1-related microarray studies of the profiles of gene expression during infection were recorded and reviewed in GEO DataSets and four studies of GSE29312, GSE29332, GSE19080 and GSE38537 were selected and analysed. In this study the people were divided into three categories: healthy individuals, asymptomatic carriers (ACs) and HAM/TSP patients. The recorded expression profiles for the Trx system, BCL2, TP53, caspase 3, caspase 8 and TNFR genes of 117 patients, including 29 healthy donors, 50 ACs and 38 HAM/ TSP patients, were evaluated. GEO2R software was used to determine differentially expressed genes (DEGs) and logarithm fold change. DEGs were calculated based on Benjamini–Hochberg false discovery rate (FDR)-adjusted *P*-value assessment and a *P*-value of less than 0.05 was considered significant. Negative LogFC values ​​indicated downregulation and positive LogFC values indicated overexpression (Table 1). Moreover, the data were analysed separately and a heatmap of the studied genes was constructed and plotted using the online server hiv-land (www.hiv.lanl.gov/content/sequence/HEATMAP/heatmap.html) (Fig. 1).

**Table 1.** List of the expression changes of the target genes in different groups

GEO studies		Trx	Trx reductase	NADPH	Scavenger ROS	BCL2	TP53	Caspase 3	Caspase 8	TNFR
GSE29312	Healthy vs ACs	0.14	−0.03	3.10	0.72	−0.27	−0.29	0.04	−0.44	−0.9
	Healthy vs HAM/TSP	0.30	0.03	−0.72	−2.11	−0.39	−0.48	0.12	−0.81	−0.20
	ACs vs HAM/TSP	−0.15	−0.07	3.17	1.93	2.17	0.18	0.73	−0.48	0.53
GSE29332	Healthy vs ACs	0.01	0.04	1.7	0.15	0.08	−0.15	−0.17	−0.20	−0.12
	Healthy vs HAM/TSP	0.66	−0.60	−2.33	0.40	−0.11	0.06	0.21	−1.06	0.16
	ACs vs HAM/TSP	−0.69	0.56	0.63	−0.56	0.05	0.88	0.14	0.72	0.19
GSE19080	Healthy vs ACs	−0.45	−0.73	0	−0.16	0.09	−0.46	0.43	0.74	0.61
	Healthy vs HAM/TSP	−0.26	−0.66	0	−0.26	0.16	−0.39	0.59	1.01	0.47
	ACs vs HAM/TSP	0.18	0.71	0	0.15	0.66	0.07	0.15	0.26	−0.14
GSE38537	Healthy vs ACs	0.72	0.15	−0.53	0.11	−0.17	0.10	−0.52	−0.48	0.46
	Healthy vs HAM/TSP	1.12	0.11	0.34	0.18	0.20	0.14	−0.24	−0.45	0.65
	ACs vs HAM/TSP	0.15	−0.04	−0.67	0.73	0.27	0.04	0.28	−0.72	0.19

### Results and Discussion

We found that Trx capacity dysregulation occurred in the HAM/TSP state. Based on the DEG results, it was found that the expression of Trx reductase and BCL2 was decreased in the HAM/TSP group compared to the AC and healthy donor groups. Further, the expression levels of Trx, scavenger ROS, TNFR (Fas) and caspase 3 in the healthy group decreased steadily compared with those in the AC and HAM/TSP patients. However, the expression levels of BCL2 and TP53, which are the most important of the genes responsible for cell survival, were increased in the AC and HAM/TSP groups compared with the healthy control group (Fig. 1).

In general, our observations revealed that the Trx system function decreases in HAM/TSP patients, whereas the expression of inducer genes responsible for cell proliferation increases. Based on the available documents, apoptosis, cell survival, tissue invasion and proliferation processes are the most important signalling pathways in HAM/TSP pathogenesis [15]. Stimulation of cell proliferation through NF_κB, TP53, AP-1 and PI3K-Akt induces the survival of HTLV-1 infected CD4+ T cells, enhancing IFN-ϒ production and stimulating the inflammation process and recruiting inflammatory cells to the CSF [15, 16]. In HAM/TSP patients, TP53 and BCL2 genes were expressed at increased levels, while caspase and TNFR (Fas) were downregulated, which in turn led to cell survival and stimulation of inflammation in these patients.

As mentioned above, free radicals, particularly reactive oxygen species, are produced during the inflammation process, which causes cell damage and cell death by triggering TNFR and caspases, but these agents are resolved by the thioredoxin redox system activities [17, 18]. According to current evidence, the thioredoxin redox system supports cell growth and inhibits apoptosis [18, 19]. In this study, we found that TNFR and caspase 3 (the main apoptotic inducers) were downregulated while BCL2 (anti-apoptotic factor) was overexpressed in HAM/TSP patients. These changes can lead to cell survival and HTLV-1 infected CD4+ T cell immortalization, which can be supported from continuous inflammation and develop into HAM/TSP and ATLL [20–22]. We also found TP53 and caspase 8 overexpression, which are known causes of cell death. TP53 and caspase 8 can induce apoptosis and can be considered for the development of novel HTLV-1 treatment approaches. According to Mulloy *et al*., HTLV-1 Tax can suppress apoptosis through the inhibition of TP53 [23]. Therefore, molecular targeting of HTLV-1 Tax efficiently enhances the overexpression of TP53 and prevents CD4+ T cell immortalization [24].

Studies about the expression changes of the genes responsible for the antioxidant function during HTLV-1 infection are quite limited. However, according to previously published reports, antioxidant capacity is reduced in HTLV-1 infection [6, 9, 12]. Shomali *et al*. have suggested that it may be appropriate to predict the progression towards the HAM/TSP by assessing the altered expression of genes responsible for the antioxidant process [6]. Corroborating previous studies, Yaghoubi *et al*. showed that the expression level of Trx system genes in HAM/TSP patients was significantly decreased compared to that in ACs and normal individuals [9]. The Trx system can reduce the progressive inflammatory process in HAM/TSP by reducing the deleterious effects of free oxygen radicals and protecting against oxidative stress due to the inflammation. It is a proper treatment alternative for HAM/TSP.

**Fig. 2.**
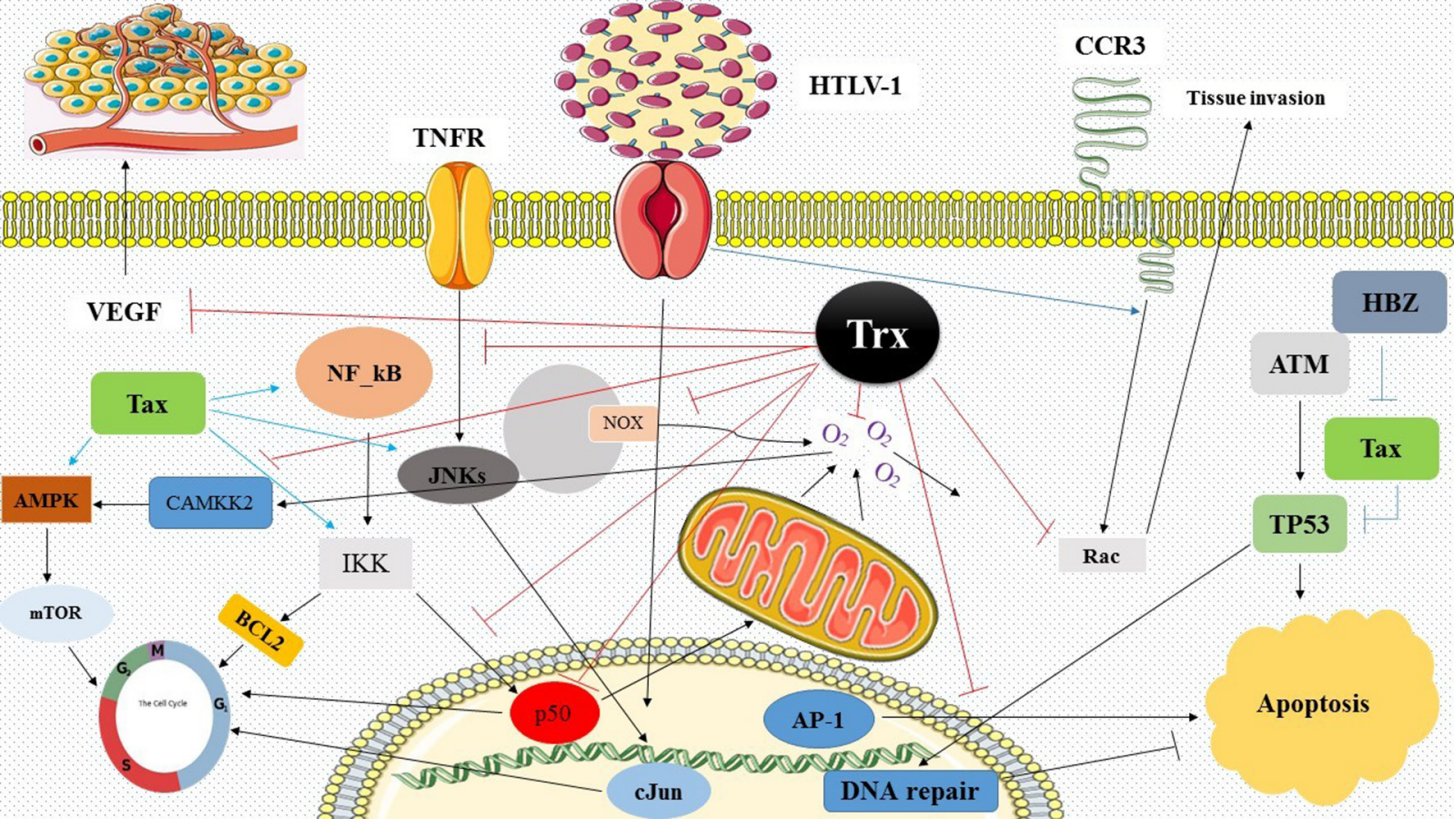
The proposed hypothetical signalling network involving a central role for Trx in HTLV-1 pathogenesis in the HAM/TSP state. In summary, Trx can contribute to HAM/TSP pathogenesis via the influence of various signalling pathways, including NF_κB, PI3K/Akt, mTOR, JNK/STAT and iNOS. We indicated that Trx can inhibit the pro-inflammatory response, tissue invasion, nitric oxide production and cell survival, which support continued inflammation in the CNS during HAM/TSP.

Finally, the molecular signalling networks based on KEGG pathway data were proposed in order to better understand the role of the Trx system during the HTLV-1 infection (Fig. 2).

According to preset results, the thioredoxin redox system inhibits the inflammatory process and cell damage with the support of anti-apoptotic biomarkers (e.g. BCL2) and through the inhibition of various inflammatory signalling pathways, including NF_κB, JNK/STAT, MAPKs, PI3K/AKT and mTOR. This efficiently reduces the inflammatory response and development into HAM/TSP. A recent clinical trial study confirmed the anti-inflammatory, immunomodulatory and anti-proliferative effects of the reducing agents when administered with IFN-α for the treatment of HAM/TSP. Therefore, the evaluation of cellular antioxidant capacity changes during HTLV-1 pathogenesis could be a new approach in the determination of the diagnostic biomarkers of HTLV-1 infection and development of a new generation of anti-HTLV-1 drugs.
